# Project Loon based augmentation for global ionospheric modeling over Southern Hemisphere

**DOI:** 10.1038/srep45976

**Published:** 2017-04-06

**Authors:** Cheng Wang, Chuang Shi, Hongping Zhang

**Affiliations:** 1 Collaborative Innovation Center for Geospatial Information Technology, No. 129 Luoyu Road, Wuhan 430079, China; 2 GNSS Research Center, Wuhan University, No. 129 Luoyu Road, Wuhan 430079, China

## Abstract

Global ionospheric products of vertical total electron content (VTEC) derived from GNSS measurements may have low accuracy over oceans and southern latitudes where there are not rich observations. Project Loon provides a great opportunity to enhance the measurements over those areas. In this contribution, a simulation of Project Loon based augmentation for global ionospheric modeling is performed by using the international reference ionosphere (IRI) which could simulate VTEC measurements for the balloons. The performance of the enhanced method based on simulation of Project Loon is investigated by comparing with VTEC maps from Ionosphere Associate Analysis Centers (IAACs) as well as IGS final GIMs. The comparison indicates that there is a better consistency between the VTEC maps by the enhanced method and IGS final GIMs. Also, obvious improvements of RMS maps in GIMs for the middle latitudes and southern latitudes are enabled by the augmentation of Project Loon. Additionally, JASON data are used to validate the specific improvement of the VTEC maps. The results show that the performance of VTEC maps is improved slightly, especially in southern latitudes. It is possible that the VTEC maps could be improved significantly by using real GPS measurements from balloons of Project Loon in the near future.

Ionospheric sounding is significant for studying the ionosphere which is an important part of the Earth’s upper atmosphere^1^. Characteristic parameters like electron density, electron temperature, total electron content (TEC) etc. can be obtained by equipment such as ionosonde, sounding rocket and radar as well as Global Navigation Satellite System (GNSS)^2^,^3^,^4^,^5^,^6^,^7^. GNSS provides an opportunity for sounding the ionosphere with high accuracy, temporal and spatial resolution. Reliable global ionosphere maps (GIM) have been generated for approximately two decade by Ionosphere Associate Analysis Centers (IAACs) of the International GNSS Service (IGS)^8^. IAACs contains the Center for Orbit Determination in Europe (CODE), the Jet Propulsion Laboratory (JPL), the European Space Agency (ESA) and the Technical University of Catalonia (Universitat Politècnica de Catalunya in Spanish, UPC) from 1998. IGS announced that three members joined IAACs in the beginning of 2016. They are Natural Resources Canada (NRCan), Chinese Academy of Sciences (CAS) and Wuhan University (WHU). These centers generate global ionospheric vertical TEC (VTEC) maps by using their own algorithm and software^9^,^10^,^11^,^12^,^13^,^14^,^15^. IGS provides the final GIMs by combination of the ionospheric products from all centers. It’s believable that the final IGS GIMs combined from more products generated by more centers would be more robust and have better precision.

Since there are a few GNSS stations installed in the ocean areas and southern latitudes, VTEC maps provided by IAACs as well as the combined final IGS GIMs could have low accuracy over these areas where there are lack of GNSS measurements. Some scholars proposed kinds of solutions to this problem. Mannucci used climatological model information as simulated data to cover the gaps between measurements^11^. Orus updated the VTEC maps using the Kriging interpolation technique and provided a better UPC GIM with an approximate 12% improvement in the self-consistency test^12^. Zhang noted there are many zero values (in fact, negative values) in ESA and CODE’s GIMs and proposed the inequality-constrained least square (ICLS) method to eliminate non-physical negative values^15^. Additionally, IRI 2012 model was utilized to provide virtual VTEC measurements over those areas where the VTEC values would be negative by original modeling^14^. So global ionospheric maps could be generated not only without negative values but also with slightly improved precision.

The essential reason of the low accuracy of VTEC over the ocean areas and southern latitudes is that there are not enough GNSS measurements over these areas. This situation could be improved if there have sufficient measurements. There are two-thirds of people without reliable Internet connection on the earth, especially in southern latitudes^16^. Google developed an innovative project “Project Loon” to provide Internet access to rural and remote areas^16^,^17^,^18^. This project uses high-altitude balloons placed in the stratosphere at an altitude of approximately 20 km to create an aerial wireless network. Starting from 2013, Google has begun some experiments in New Zealand, Brazil and South Africa, etc. It should be pointed out that each balloon is equipped with a GPS receiver for tracking its location. If the GPS measurements could be provided by Google, the global ionospheric modeling might be enhanced over the ocean areas and southern latitudes. In this manuscript, an augmentation for global VTEC modeling based on Project Loon is proposed. The first section of the manuscript will present the principle methodology of global ionospheric modeling along with the augmentation base on Project Loon. Additionally, the performance of the augmentation is investigated by comparison of VTEC maps between our ionospheric products and GIMs from other four IAACs. Furthermore, a validation of the augmentation against external independent JASON data is carried out. Finally, conclusions are summarized in the last section.

## Basic methodology of global VTEC modeling

### GIMs derived from GPS measurements

IAACs compute global ionospheric VTEC maps independently using different approaches. CODE uses a spherical harmonic (SH) expansion referring to a solar geomagnetic frame for representing GIMs^13^. JPL’s approach is based on interpolating TEC within triangular tiles and has been extended to include climatological models as simulated data, so that VTEC maps can be generated without gaps^11^. ESA developed a three-dimensional mathematical ionosphere model based on a simple Chapman profile approach, and later used SH expansion instead^19^. UPC used a two-layer tomographic model for the TEC estimation and then present improving GIMs by using Kriging interpolation technique^12^. In this paper, the spherical harmonic functions used for global ionospheric modeling are the same as those used by CODE and ESA. The basic equations are presented for ionosphere modeling, as follows:


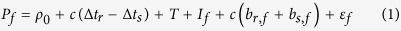


where the subscript *f* indicates the frequency dependency of the terms; *P* is the code measurements; *ρ*_0_ is the geometric range between the receiver and a satellite; *c* is the speed of light; Δ*t*_*r*_ and Δ*t*_*s*_ are the respective clock errors of receiver and satellite with respect to GPS time; *T* is the tropospheric delay; *I* is the ionospheric delay; *b*_*r*_ and *b*_*s*_ are the respective hardware delays of receiver and satellite; and *ε* contains the multipath effect, measurement noise, and other error sources. Code measurements are smoothed by the carrier-phase measurements to obtain high-precision code observables. The non-dispersive terms are eliminated by the difference between the carrier-smoothed code measurements, as shown in Eq. (2):


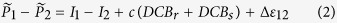


where 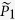,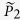 are the smoothed code measurements; *DCB*_*r*_ and *DCB*_*s*_ are the differential code bias (DCB) of receiver and satellite, respectively. The widely used thin shell approximation of the ionosphere is followed. The mapping function MLSM is used to transform Slant TEC to VTEC^13^. Ignoring the noise term, Eq. (2) can be re-written as Eq. (3), where *mf* is the ionospheric mapping function, which depends on the zenith distance *z* at the station, and VTEC is the vertical TEC at the ionospheric pierce point (IPP).


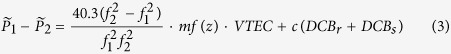


An SH function is used for modeling VTEC referring to a solar geomagnetic frame as the following Eq. (4)^13^, where *φ* is the geomagnetic latitude of IPP; *λ* is the sun-fixed longitude of IPP; *n* and *m* are the degree and order of the model, respectively; 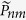 is the normalized associated Legendre function of degree *n* and order *m*; and *a*_*nm*_ and *b*_*nm*_ are the unknown SH coefficients and GIM parameters, respectively.


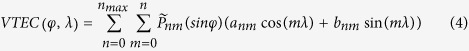


In this work, GPS measurements of approximately 325 IGS stations are used for modeling, and a minimum elevation cutoff of 20° is applied to avoid particularly noisy measurements. VTEC modeling is in a solar-geomagnetic reference frame using spherical harmonic expansions up to a degree and order of 15. The SH coefficients are considered to vary linearly with time, which means that the parameters are linearly interpolated between consecutive nominal epochs. Additionally, one-day GPS data are divided into 12 sessions, and each session contains two hours of measurements. The DCB of satellites and receivers will be estimated along with the SH coefficients, where a DCB datum is defined by a zero-mean condition imposed on all of the satellite biases.

### Project Loon based augmentation

Project Loon is Google’s pursuit to deploy a high-altitude balloon network operating in the stratosphere, at an altitude of about 18 km^20^. This particular layer of the stratosphere is advantageous because of its relatively low wind speeds and minimal turbulence^20^. By moving with the wind, the balloons can be arranged to form a large communication network. The location of each balloon is tracked by a GPS receiver. Since the balloons are deployed at approximately 20 km altitude, the GPS receiver could observe the electron densities from the bottom of ionosphere to the orbit height of the GPS satellite. So, the TEC obtained by the balloons is similar with the ground-based GPS receivers.

**Figure 1:**
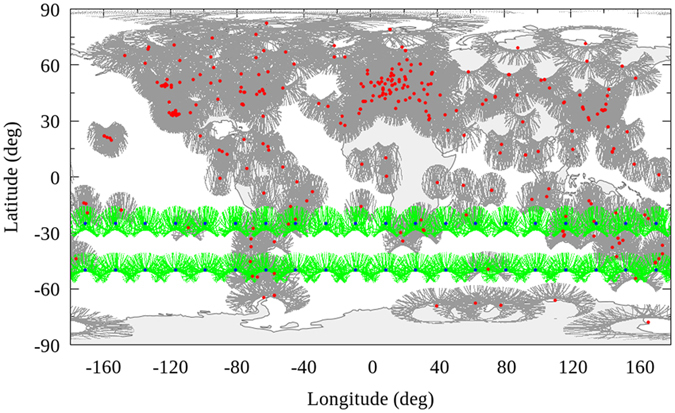
Map showing the geographical distribution of IGS stations, 40 balloons and IPPs. The red and blue dots present the distribution of IGS stations and balloons, respectively. The grey and green lines depict the IPPs observed by ground-based stations and Project Loon balloons, respectively. This figure is drawn using Gnuplot V5.0 (http://gnuplot.sourceforge.net) with map data of the world from Natural Earth (http://www.naturalearthdata.com/downloads/).

Google has done several pilot experiments in New Zealand, Brazil and South Africa, etc. since 2013^20^. Some experiments have made successful, while some balloons crashed. Since Project Loon is still in the experimental stage, the augmentation for global ionospheric modeling based on Loon balloons could be simulated by using existed empirical models such as IRI model or NeQuick. In this study, 40 balloons are supposed to be deployed at the 25^th^ and 50^th^ parallel south. There are 20 balloons at each parallel south. Figure 1 shows the global geographical distribution of approximately 325 IGS stations, balloons and the ionospheric pierce points (IPPs) during a 24-hour period. In the simulation of Project Loon based augmentation, the location of each balloon is supposed to be confirmed. So each balloon could be considered as a station like IGS stations. The only difference is that IGS stations are installed on the ground in contrast to the balloons being deployed in the stratosphere. These balloons could also observe the GPS satellites and get the ionospheric TEC. And in this study, the VTEC values at IPP observed by the balloons are simulated by IRI model.

IRI is the internationally recognized empirical model. It was developed and improved by a joint work group of the Committee on Space Research (COSPAR) and the International Union of Radio Science (URSI)^21^,^22^,^23^. The IRI-TEC value is the integral of the electron density profile derived from the IRI 2012 model. As shown in Fig. 1, the VTEC values obtained by balloons are simulated through empirical model IRI 2012. Since IRI is a climatological model, it cannot provide TEC values with the high accuracy as GPS-TEC values. Many authors have reported evaluations of the IRI model over different areas of the world during periods of quiet or active space weather^24^,^25^,^26^,^27^,^28^,^29^,^30^,^31^,^32^,^33^,^34^,^35^,^36^,^37^. The capabilities of and improvements to the IRI model have been shown, but there is not very good agreement between the IRI model predictions and the observed values, especially during periods of active space weather. Many authors have reported evaluations of the IRI model using some ionospheric characteristics parameters like TEC over many areas of the world during quiet or active space weather^25^,^29^,^33^,^34^,^38^. Also, in a previous study of the authors, the investigation shows that the RMS values of the differences between IRI predicted TEC values and IGS GIMs could range from 10 to 18 TECU^14^. They have shown the abilities and improvements of the IRI model to predict ionospheric TEC in recent years. Since the IRI model only provides a profile of up to an altitude of 2000 km, there is an additional part of the TEC above the 2000 km altitude. Therefore, the IRI-TEC values shouldn’t be used to combine with observed GPS-TEC values for modeling directly. In this study, IRI-TEC values are supposed to have a bias referring to GPS-TEC values. The daily bias between IRI-TEC and GPS-TEC could be considered to be an unknown parameter and be estimated along with SH coefficients as well as DCBs of satellite and receivers. Also, IRI-TEC and GPS-TEC values are considered to have different weights in the adjustment. The weights of GPS-TEC values usually be confirmed by elevation angle of each satellite measurement. A priori standard deviation of IRI-TEC values is assumed to be 15 TECU because the IRI predictions may not represent the real status of the ionosphere, especially during active space weather. Then the weights could be determined by the law of variance covariance propagation^39^.

## Results and Discussion

### Test and reference data

GPS measurements observed by approximately 325 IGS stations during the day of year (DOY) 152~181 in 2015 are collected for global ionospheric VTEC modeling. The assessment of proposed method is carried out by the comparison of VTEC values among the daily ionospheric products by our solutions, IAACs’ products and the final IGS combined GIMs. The comparison could be depicted in terms of the bias and root mean square (RMS) of the differences between the two ionospheric products, as shown in Eqs (5) and (6), where *n* is the total number of VTEC values in daily GIM product; *VTEC*_*a*_ and *VTEC*_*b*_ are VTEC values from two products of GIMs which could be our WHU products, or IAACs’ products and IGS GIMs; *a* indicates that the VTEC value from one product; *b* indicates that the VTEC value from another product. The term bias is basically means the central tendency of a set particular set of data. In this study, the bias is actually described by an arithmetic mean of the difference between VTEC maps in two ionospheric products. However, the bias doesn’t tell much about the difference of two data set. Because the negative values cancel the positive values, probably leaving an average of zero. If we want to know the magnitude of the difference without regard for positive or negative, the RMS calculation as Eq. (6) presented could make the values all positive and take the square root of the average. In this study, the bias could show whether there is a remarkable systematic error between two ionospheric products in general. Moreover, RMS values could be used to describe the magnitude of the difference between two ionospheric products. The higher RMS value indicates that the difference is larger.


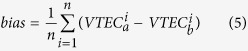



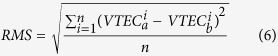


The IONEX is an internationally adopted format for the exchange of two dimensional ionosphere maps. The IONEX format gives definition of the grid in latitude from −87.5 to 87.5 with increment of 2.5 in degrees, and in longitude from −180 to 180 with increment of 5 in degrees. Each VTEC map contains a number of 71 × 73 grids. And there are 13 maps of VTEC values in a daily GIM product. Thus, the total number of VTEC values for a daily GIM product is equal to 67379 (71 × 73 × 13).

Furthermore, external independent VTEC measurements observed by JASON satellite are used to validate those VTEC values in GIMs which derived from GPS measurements. JASON VTEC measurements have been used to validate final IGS ionospheric products^8^. While, it should be noted that GNSS-derived VTEC includes the plasmaspheric electron content contribution which is typically 10% during daytime and up to 60% at night, in contrast to JASON VTEC covering from bottom of ionosphere to JASON orbit at an altitude of approximately1300 km of electron densities. Although JASON VTEC measurements are affected by an offset with respect to GNSS-derived VTEC values, they are very accurate for validation the performance of GIMs over the oceans.

### Improvement of RMS maps

**Figure 2:**
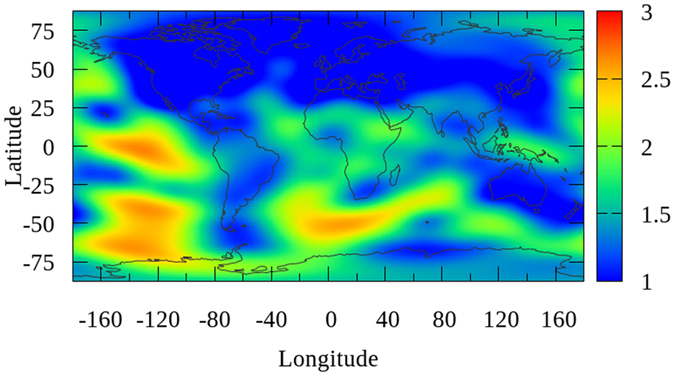
Map showing the average RMS values by the original solution for the 30 day period from DOY 152–181, 2015. The scale of this map is 1 TECU to 0.1 TECU. This figure is drawn using Gnuplot V5.0 (http://gnuplot.sourceforge.net) with map data of the world from Natural Earth (http://www.naturalearthdata.com/downloads/).

**Figure 3:**
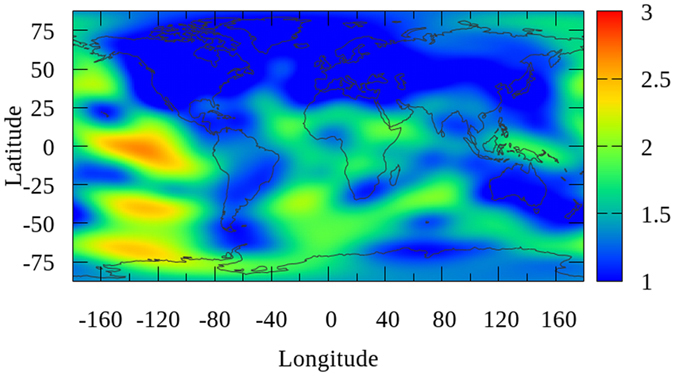
Map showing the average RMS values by the proposed solution based on Project Loon augmentation for the 30 day period from DOY 152–181, 2015. The scale of this map is 1 TECU to 0.1 TECU. This figure is drawn using Gnuplot V5.0 (http://gnuplot.sourceforge.net) with map data of the world from Natural Earth (http://www.naturalearthdata.com/downloads/).

The daily global ionospheric products for DOY 152–181 in 2015 are generated by using the GPS measurements of approximately 325 IGS stations. The modeling are performed through the original solution and proposed method based on Project Loon, respectively. RMS maps are generated along with global VTEC maps, which indicate the internal coincident precision of VTEC value at each grid in GIMs. The average of RMS maps by the two solutions for the 30 day period from DOY 152–181, 2015 are presented as shown in Figs 2 and 3 (unit: 0.1 TECU), respectively. The RMS presented in these two figures are calculated by the average of 30 values for each grid during the 30 day period. According to the RMS values presented in Figs 2 and 3, the precision of VTEC values over continents are much higher than those over the Pacific, Atlantic, and Indian Oceans, Africa and the polar region. Also, as depicted in Fig. 3, the average RMS map is apparently improved over the southern latitudes. In order to investigate the specific improvement, the globe is divided into 3 latitudinal bands, the Northern Band (32.5°~87.5°), the Middle Band (−32.5°~32.5°) and the Southern Band (−87.5°~–32.5°). The average of RMS values for the globe and the 3 bands are calculated individually and are shown in Table 1 (unit: 0.1 TECU). From the values in Table 1, the internal coincident precision of the VTEC map for the Northern Band is the highest among the 3 bands, followed by the Middle Band and the Southern Band. The average of RMS values shows a very slight improvement for Northern Band. Because the simulation of Project Loon in this study has no balloons in northern latitudes. In contrast, since Project Loon balloons providing additional GPS measurements over Southern Hemisphere, there are obviously improvements for the Middle Band and Southern Band, obtaining a 1.40% and 8.54% improvement, respectively. The internal coincident precision of the global VTEC maps is improved by approximately 3.97%. These results indicate that VTEC RMS maps are substantially improved through Project Loon based augmentation.

### Comparison of VTEC maps

**Figure 4:**
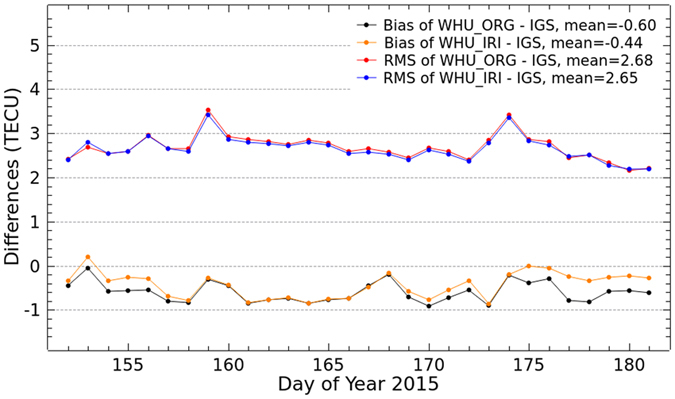
Bias and RMS values from the comparison of VTEC maps by original solution and Project Loon based augmentation solution and the IGS final GIMs for DOY 152–181 in 2015.

**Figure 5:**
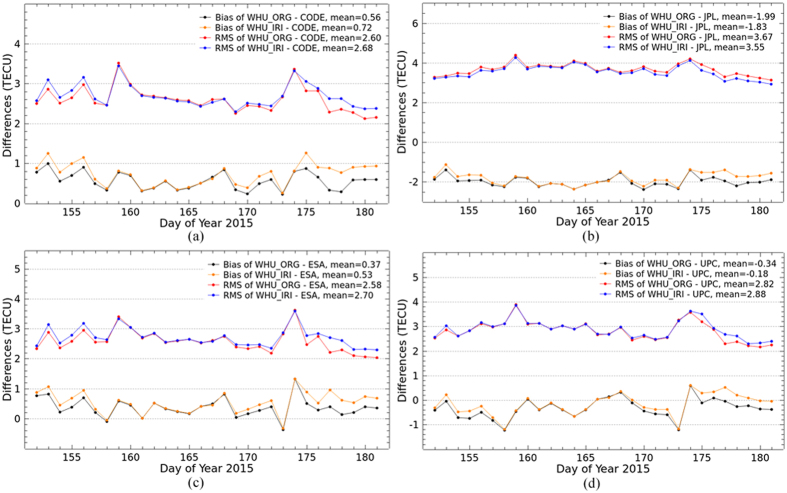
Bias and RMS values from the comparison of VTEC maps by original solution and Project Loon based augmentation solution and GIMs from IAACs for DOY 152–181 in 2015.

VTEC maps generated by the two solutions are now further compared with the IGS final combined products and the products from IAACs for DOY 152–181, 2015. IGS has been providing reliable ionospheric products by combining GIMs produced by CODE, JPL, ESA and UPC since 1998. In this study, the two kinds of VTEC maps by the original solution and the proposed Project Loon augmented solution are compared with GIMs from CODE, JPL, ESA and UPC and the IGS final combined GIMs. As shown in Figs 4 and 5, the bias and RMS values of the differences are calculated between our VTEC maps and the IGS final GIMs as well as the products from IAACs. In the figures, WHU_ORG and WHU_IRI represent the VTEC maps by the original solution and the Project Loon augmented solution using simulation with IRI 2012 model, respectively. In Fig. 4, the black and orange dotted lines show the bias of differences between WHU_ORG and WHU_IRI and the IGS final GIMs, respectively. The red and blue dotted lines show the RMS values of differences between WHU_ORG and WHU_IRI and the IGS final GIMs, respectively. The bias and RMS values are presented by dotted lines with the same color in Fig. 5 to show the differences between WHU products and GIMs from IAACs.

As shown in Fig. 4, approximately half of bias between WHU_IRI and IGS GIMs are more closely to zero, compared to that between WHU_ORG and IGS GIMs. Also, most RMS values of the differences between WHU_IRI and IGS GIMs are very slight smaller than those between WHU_ORG and IGS GIMs, except on the DOY 153. These bias and RMS values indicate that there is a better consistency between WHU_IRI and IGS GIMs. Although the improvement looks limited, the simulation with IRI model of Project Loon based augmentation is effective. Figure 5 presents a similar situation of bias between WHU products and GIMs from IAACs. Compared to the original solution, RMS values of the differences between WHU_IRI and GIMs from CODE, ESA and UPC are a little larger on several days. While, most RMS values of the differences between WHU_IRI and JPL GIMs are slightly smaller. Also, the bias of WHU_IRI and JPL GIMs are more closely to zero. These results indicate there is a better consistency between WHU_IRI and JPL GIMs. In contrast, the consistencies between WHU_IRI and GIMs from CODE, ESA and UPC seem to become worse. This does not mean that the proposed method of augmentation for global ionospheric modeling based on Project Loon is not feasible.

**Figure 6:**
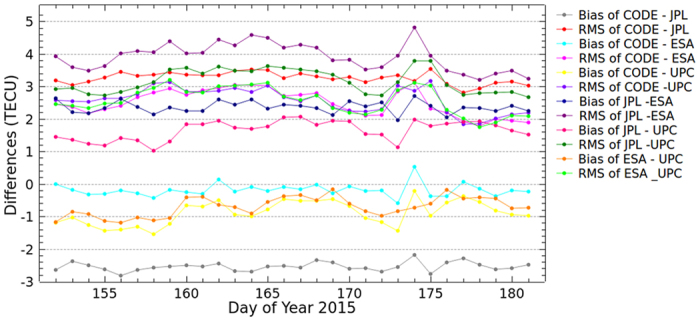
Bias and RMS values of the differences among the GIMs from IAACs for DOY 152–181 in 2015.

As mentioned above, there are a few GPS measurements over the oceans and southern latitudes. At present, only GNSS measurements are used by all centers of IAACs for global ionospheric VTEC modeling. Different data processing strategies are used by IAACs for dealing the gap of GNSS measurements over the oceans and southern latitudes. CODE and ESA set the VTEC values which is negative by original SH modeling to zero directly in GIM. UPC used Kriging interpolation technique to improve the accuracy of VTEC values over the areas where there could be lack of GPS measurements. Additional simulated data based on climatological model are covered the gap between measurements by JPL. The bias and RMS values of the differences among the GIMs from IAACs are shown in Fig. 6. Also, the average of bias and RMS values for DOY 152–181, 2015 are presented in Table 2. According to Fig. 6 and Table 2, the bias are approximately plus or minus 3 TECU; the RMS values range from 2 to 5 TECU. Also, Figs 4 and 5 showing the differences between WHU products and GIMs from IACCs indicate that WHU products have the same level of performance as the products of IAACs. Different data processing strategies might be the main reason of the differences among the GIMs provided by different centers. That also could explain the differences between WHU products and GIMs from IAACs, even the RMS values becoming larger when the Project Loon based augmentation is performed in the modeling.

**Figure 7:**
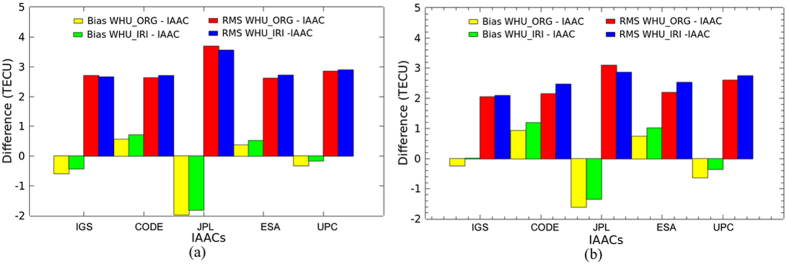
Bias and RMS values of the differences between WHU products and IGS GIMs as well as IAACs’ products during DOY 152–181, 2015.

Since the balloons are supposed to be deployed at the 25^th^ and 50^th^ parallel south, it is necessary to investigate the performance of the augmentation over southern latitudes. According to the geographical distribution of IPPs by the augmentation in Fig. 1, the VTEC values covering from 20°S to 60°S are collected for the investigation. Figure 7 shows that the bias and RMS values of the differences over the globe and southern latitudes (20°S–60°S) between WHU products and IGS GIMs as well as IAACs’ products during DOY 152–181, 2015, respectively. Also, the differences over the specified southern latitudes among the IAACs’ products are presented in Table 3. Firstly, according to RMS values, the magnitude of differences over globe is apparently larger than that over the specified southern latitudes. Because there are more differences of VTEC maps over other regions besides specified southern latitudes. Also, the bias and RMS values for global comparison have changed little by the augmented solution. However, for the specified southern latitudes, there are an obvious change after taking the augmentation of Project Loon. Additionally, RMS value of the differences between WHU_IRI and JPL GIMs are a little smaller. While, the RMS values between WHU_IRI and GIMs from CODE, ESA and UPC seem to become larger. It doesn’t indicate that the modeling with Project Loon based augmentation would have no improvement. The explanation could be referred to the previous paragraph.

### Latitudinal performance

**Figure 8:**
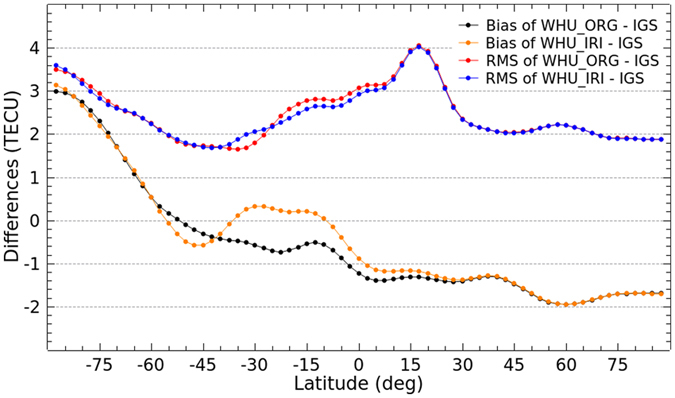
Bias and RMS values in latitudes from the comparison of VTEC maps by original solution and Project Loon based augmentation solution and the IGS final GIMs for DOY 152–181 in 2015.

**Figure 9:**
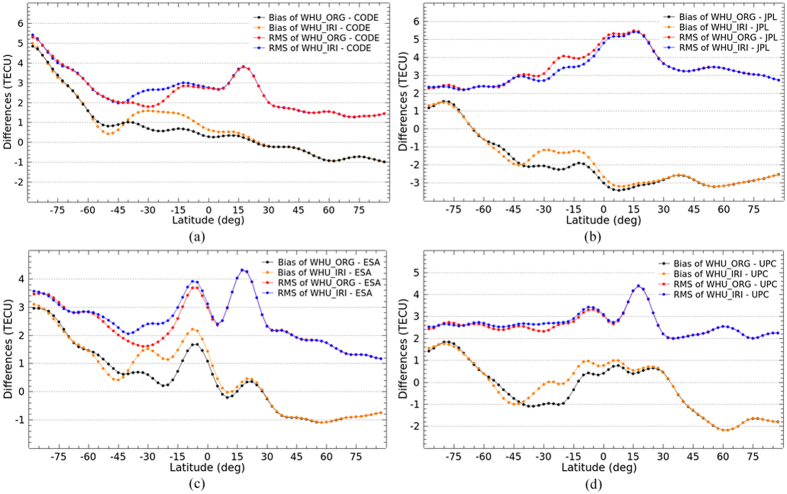
Bias and RMS values in latitudes from the comparison of VTEC maps by original solution and Project Loon based augmentation solution and GIMs from IAACs for DOY 152–181 in 2015.

The latitudinal performance of the enhanced ionospheric products based on simulation of Project Loon is also investigated. It is analyzed by comparing VTEC values in the same latitude with the IGS final GIMs as well as the GIMs from IAACs. As shown in Figs 8 and 9, the bias and RMS values of the differences of VTEC maps in latitudes between WHU products and IGS final GIMs as well as GIMs from IAACs are presented. As depicted in Fig. 8, there is an obvious change in the differences, especially the bias in southern latitudes. The bias between WHU_IRI and IGS GIMs are more closely to zero compared to WHU_ORG. Also, RMS values of the differences between WHU_IRI and IGS GIMs are very slight smaller than those between WHU_ORG and IGS GIMs in low latitudes. Figure 9(b) shows a similar situation of bias and RMS values of the differences between WHU products and GIMs from JPL. In contrast to before augmentation, the differences between WHU_IRI and GIMs from CODE, ESA and UPC become larger in terms of bias and RMS values presented in Fig. 9, especially in low and middle latitudes of Southern Hemisphere. The differences of VTEC maps depicted in the previous section for DOY 152–181, 2015 mainly come from the differences in southern latitudes where there are augmented measurements based on Project Loon with a simulation using IRI model.

Although the differences between WHU_IRI and GIMs from three centers (CODE, ESA and UPC) are larger, it doesn’t mean that the WHU_IRI gives lower accuracy of VTEC maps. Fortunately, the differences between WHU_IRI and IGS final GIMs become smaller compared to the original solution. The consistency of WHU_IRI and IGS final GIMs is better, but not much. On the one hand, the augmented measurements in southern latitudes are simulated by using IRI model which gives relative low accuracy of VTEC values. On the other hand, there are still some gaps of measurements over oceans and southern latitudes even an augmentation based on Project Loon is performed. Even if the differences between WHU_IRI and IGS final GIMs become larger, it is possible that the WHU_IRI provides better VTEC values compared to the GIMs by WHU_ORG. Because the IGS final GIMs are combined by using the VTEC maps from IAACs which use only GNSS measurements for the modeling. Since there are not rich data of GNSS measurements over the oceans and southern latitudes, the accuracy of IGS final GIMs over those areas might be not high as that in northern latitudes.

### Validation with JASON data

**Figure 10:**
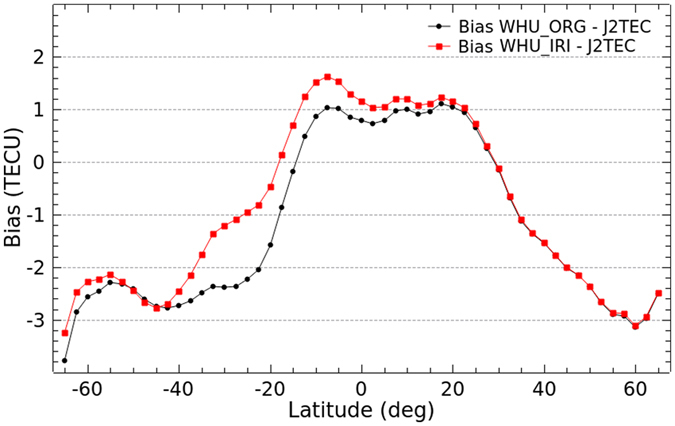
Bias of the differences between WHU products and J2TEC for DOY 152–181 in 2015.

**Figure 11:**
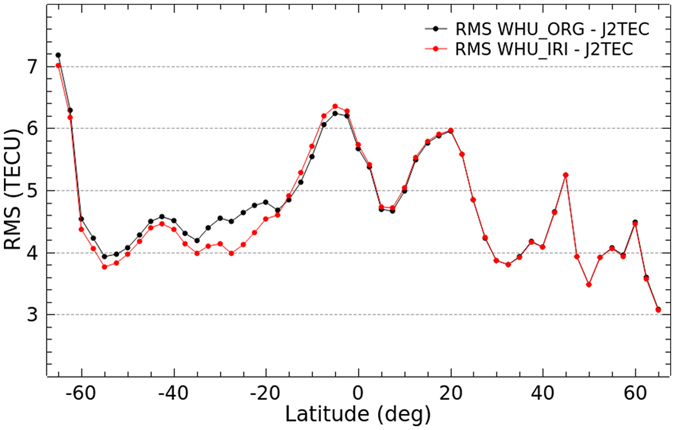
RMS values of the differences between WHU products and J2TEC for DOY 152–181 in 2015.

As an external independent VTEC measurements, JASON data is very useful for evaluation of the accuracy or specific improvement of the VTEC maps based on Project Loon augmentation over oceans and southern latitudes. Unless otherwise indicated, VTEC measurements from JASON 2 thereafter is referred to as J2TEC. Figure 10 and 11 show bias and RMS values of the differences in latitudes between WHU products (WHU_ORG and WHU_IRI) and J2TEC for the 30 day period from DOY 152–181, 2015, respectively.

As the bias shown in Fig. 10, absolute values of bias between WHU_IRI and J2TEC in most southern latitudes are apparently smaller than those between WHU_ORG and J2TEC. Compared to WHU_ORG, the bias between WHU_IRI and J2TEC is larger in low latitudes of both Northern Hemisphere and Southern Hemisphere. Although the balloons of Project Loon are supposed to be deployed at the 25^th^ and 50^th^ parallel south, it’s possible that the VTEC values in GIMs near the 25^th^ and 50^th^ parallel south might be affected by the augmentation. The modeling actually is a process of fitting of parameters using the measurements. And the VTEC values could be represented by the linear combination of the estimated parameters. Since the modeling with an augmentation by Project Loon, the represented VTEC values are more or less different from the original values. Moreover, RMS values of the differences between WHU products and J2TEC are presented in Fig. 11. The RMS values decrease obviously in the southern latitudes from −60° to −15°, especially near the 25^th^ parallel south. Also, there is a very slight increase of RMS values in low latitudes of Southern Hemisphere. Overall, the performance of VTEC maps by the augmentation based on Project Loon is better than those by the original solution. Since the augmentation is performed through simulation of VTEC measurements by using the IRI model, the improvement is not much. It is believable that the improvement of VTEC maps could be significant when the real GPS measurements observed by the balloons of Project Loon are collected and used for the modeling.

**Figure 12:**
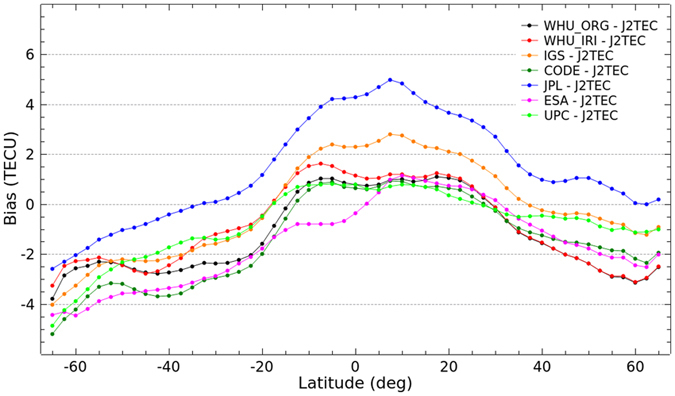
Bias of the differences between GIMs from IAACs and J2TEC for DOY 152–181 in 2015.

**Figure 13:**
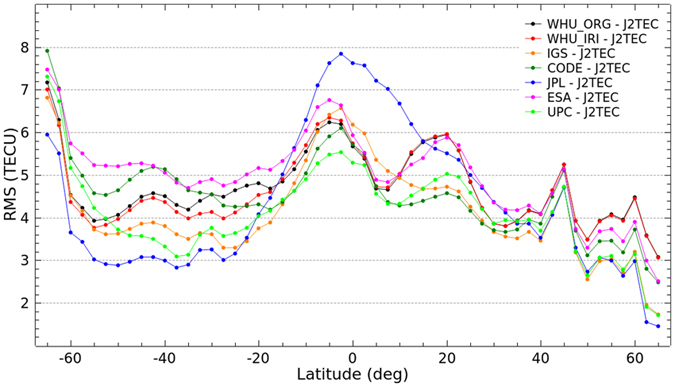
RMS values of the differences between GIMs from IAACs and J2TEC for DOY 152–181 in 2015.

Additional validation of VTEC maps from IAACs as well as IGS final GIMs is performed with respect to JASON data. Figure 12 and 13 show the bias and RMS values of the differences between GIMs and J2TEC, respectively. The bias shows a similar trend as presented in Fig. 12. According to the bias, most VTEC values of GIMs from CODE, ESA, UPC, WHU and IGS are smaller than J2TEC, especially in middle and high latitudes in contrast to larger than J2TEC in low latitudes. While, VTEC values from JPL mostly are larger than J2TEC in northern latitudes and low southern latitudes. The absolute values of bias in southern latitudes are larger than those in northern latitudes, especially in mid-high latitudes. It indicates once more that the accuracy of VTEC maps in southern latitudes is lower. The RMS values presented in Fig. 13 also could depict this point. Because the RMS values are apparently larger in southern latitudes, especially in high latitudes. Also, the dispersion of RMS values in southern latitudes are in a relatively larger area. Since there are not rich GNSS measurements in southern latitudes, IAACs provide their quite different accuracy of VTEC values over Southern Hemisphere. These comparative results also indicate that WHU products have the same level of performance as the VTEC maps provided by IAACs. Overall, the IGS final combined GIMs is more stable and more accurate than GIMs provided by each center.

## Conclusions

More and more IGS stations have been installed on the globe over a decade ago. Most stations are located on the continents, and a few in the oceans and Southern Hemisphere. There are not rich GNSS measurements in southern latitudes. It might be the main reason of the low accuracy of VTEC maps over those areas. Google started Project Loon to provide Internet access to rural and remote areas a few years ago. Balloons equipped with a GPS receiver are deployed in the stratosphere at an altitude of approximately 20 km. It could be a great opportunity to improve the VTEC maps over southern latitudes by using the GPS measurements observed by those balloons. A simulation of Project Loon based augmentation for global ionospheric modeling is performed by using IRI model. The investigation shows that there are obvious improvements of RMS maps in GIMs for the Middle Band and Southern Band, obtaining a 1.40% and 8.54% improvement, respectively. The performance of the augmented products is investigated by comparison of VTEC maps and GIMs from IAACs. The comparison indicates that there is a better consistency between the VTEC maps by the enhanced method based on Project Loon and IGS final GIMs. Also, a validation is carried out to investigate the specific improvement of the VTEC maps by using JASON data. The results show that VTEC maps are improved slightly over southern latitudes. Moreover, VTEC maps from IAACs and IGS final GIMs are validated with respect to JASON VTEC measurements. IAACs provide quite different accuracy of VTEC values in southern latitudes where there are not rich GNSS measurements. VTEC maps from WHU have the same level of performance as the GIMs provided by IAACs. IGS final GIMs have best performance among the GIMs from IAACs in terms of bias and RMS values. In general, the performance of VTEC maps enhanced by Project Loon with a simulation by IRI model is improved slightly. The global ionospheric VTEC maps have the potential significant improvement by using the real GPS measurements observed by the balloons of Project Loon in the near future.

## Additional Information

**How to cite this article:** Wang, C. *et al*. Project Loon based augmentation for global ionospheric modeling over Southern Hemisphere. *Sci. Rep.* **7**, 45976; doi: 10.1038/srep45976 (2017).

**Publisher's note:** Springer Nature remains neutral with regard to jurisdictional claims in published maps and institutional affiliations.

## Figures and Tables

**Table 1:** Average of the RMS maps for the 30 day period from DOY 152–181, 2015 (unit: 0.1 TECU).

	North Band	Middle Band	South Band	All
Original	1.078	1.496	1.733	1.436
Augmented	1.077	1.475	1.585	1.379
Improvement	0.09%	1.40%	8.54%	3.97%

**Table 2:** The average of bias and RMS values of the differences among the GIMs from IAACs for DOY 152–181, 2015.

	Bias			RMS		
	JPL	ESA	UPC	JPL	ESA	UPC
CODE	−2.54	−0.19	−0.89	3.26	2.50	2.58
JPL	—	2.35	1.65	—	3.90	3.16
ESA	—	—	−0.70	—	—	2.57

(Unit: TECU).

**Table 3:** The bias and RMS values of the differences over southern latitudes among the GIMs from IAACs during DOY 152–181, 2015.

	Bias			RMS		
	JPL	ESA	UPC	JPL	ESA	UPC
CODE	−2.55	−0.19	−1.56	3.25	2.21	2.89
JPL	—	2.37	0.99	—	3.67	2.42
ESA	—	—	−1.38	—	—	2.94

(Unit: TECU).
